# Predicting in-hospital all-cause mortality in heart failure using machine learning

**DOI:** 10.3389/fcvm.2022.1032524

**Published:** 2023-01-11

**Authors:** Dineo Mpanya, Turgay Celik, Eric Klug, Hopewell Ntsinjana

**Affiliations:** ^1^Division of Cardiology, Department of Internal Medicine, School of Clinical Medicine, Faculty of Health Sciences, University of the Witwatersrand, Johannesburg, South Africa; ^2^Wits Institute of Data Science, University of the Witwatersrand, Johannesburg, South Africa; ^3^School of Electrical and Information Engineering, Faculty of Engineering and Built Environment, University of the Witwatersrand, Johannesburg, South Africa; ^4^Netcare Sunninghill, Sunward Park Hospitals and Division of Cardiology, Department of Internal Medicine, School of Clinical Medicine, Faculty of Health Sciences, University of the Witwatersrand, Johannesburg, South Africa; ^5^Department of Paediatrics and Child Health, School of Clinical Medicine, Faculty of Health Sciences, University of the Witwatersrand, Johannesburg, South Africa

**Keywords:** machine learning, heart failure, mortality, predict, Africa

## Abstract

**Background:**

The age of onset and causes of heart failure differ between high-income and low-and-middle-income countries (LMIC). Heart failure patients in LMIC also experience a higher mortality rate. Innovative ways that can risk stratify heart failure patients in this region are needed. The aim of this study was to demonstrate the utility of machine learning in predicting all-cause mortality in heart failure patients hospitalised in a tertiary academic centre.

**Methods:**

Six supervised machine learning algorithms were trained to predict in-hospital all-cause mortality using data from 500 consecutive heart failure patients with a left ventricular ejection fraction (LVEF) less than 50%.

**Results:**

The mean age was 55.2 ± 16.8 years. There were 271 (54.2%) males, and the mean LVEF was 29 ± 9.2%. The median duration of hospitalisation was 7 days (interquartile range: 4–11), and it did not differ between patients discharged alive and those who died. After a prediction window of 4 years (interquartile range: 2–6), 84 (16.8%) patients died before discharge from the hospital. The area under the receiver operating characteristic curve was 0.82, 0.78, 0.77, 0.76, 0.75, and 0.62 for random forest, logistic regression, support vector machines (SVM), extreme gradient boosting, multilayer perceptron (MLP), and decision trees, and the accuracy during the test phase was 88, 87, 86, 82, 78, and 76% for random forest, MLP, SVM, extreme gradient boosting, decision trees, and logistic regression. The support vector machines were the best performing algorithm, and furosemide, beta-blockers, spironolactone, early diastolic murmur, and a parasternal heave had a positive coefficient with the target feature, whereas coronary artery disease, potassium, oedema grade, ischaemic cardiomyopathy, and right bundle branch block on electrocardiogram had negative coefficients.

**Conclusion:**

Despite a small sample size, supervised machine learning algorithms successfully predicted all-cause mortality with modest accuracy. The SVM model will be externally validated using data from multiple cardiology centres in South Africa before developing a uniquely African risk prediction tool that can potentially transform heart failure management through precision medicine.

## Highlights

- Models predicting outcomes in heart failure are built mainly from data originating in high-income countries, where ischaemic heart disease predominates.
- In this study, we used data of in-hospital heart failure patients to train machine learning algorithms to predict all-cause mortality.
- This is the first sub-Saharan African model demonstrating machine learning algorithms’ utility in making predictions.
- In addition, the study shows that the performance of machine learning algorithms is sensitive to the sample size and the credibility of the data used to train algorithms.

## 1. Introduction

Patients diagnosed with heart failure experience a high mortality rate, with 30–50% of patients demising within 5 years from diagnosis (1). This is despite the introduction of novel pharmacological and intracardiac device therapy. Furthermore, due to the high cost associated with the implantation of device therapy and transplant services, only a select few patients in sub-Saharan Africa (SSA) can access these life-saving interventions. As such, there is a need to risk stratify patients with precision, ensuring that the right patient receives the right therapy at the right time.

Risk calculators are derived from predictive modeling, and they are used to estimate the risk of outcomes such as mortality and rehospitalisation in heart failure. Most readily available risk calculators were created using data predominantly from high-income countries (HIC). For example, the Meta-Analysis Global Group in Chronic Heart Failure (MAGGIC) risk calculator, created from a statistical Poisson model, estimates the risk of one and 3-year mortality in heart failure (2). Another widely available risk calculator predicting in-hospital all-cause mortality was derived from the Get with the Guidelines Heart Failure data of multiple sites in HIC, using a multivariable logistic regression model (3). To the best of our knowledge, risk calculators derived from SSA do not exist, and existing heart failure predictive models have not been validated using data originating from SSA.

Predictive models are population-specific and are prone to inherent biases such as the availability of therapy, referral pathways, genotype, and varying population data used to create these models. Furthermore, most of the available risk calculators were derived using data from heart failure patients in whom the primary cause of heart failure is ischaemic heart disease, unlike in SSA, where the predominant cause of heart failure is non-ischaemic heart disease. Therefore, there is a need to create uniquely African predictive models, mainly using machine learning techniques, since machine learning algorithms are capable of learning from a labelled dataset prior to making predictions. Also, the accuracy of predictions may improve over time as the algorithms are exposed to a larger dataset. This study aims to use machine learning algorithms to predict all-cause mortality in heart failure patients hospitalised in a tertiary academic centre in Johannesburg, South Africa.

## 2. Materials and methods

### 2.1. Study design and participants

Patient data was exported from the PMRCardio database, a Microsoft Structured Query Language Server Management Studio version 15.0.18330.0, that stores clinical data of patients hospitalised in cardiology wards. The Charlotte Maxeke Johannesburg Academic Hospital (CMJAH) is a tertiary-level state-owned institution equipped with general cardiac wards, a cardiac intensive unit, a catheterisation and electrophysiological laboratory, and outpatient clinics.

All acutely and chronically ill patients with cardiovascular diseases are hospitalised in dedicated cardiac wards. Their admission data is stored digitally in the PMRCardio database by trainee physicians rotating in the cardiology wards. Clinical parameters available in the dataset (features) include demographic data, clinical history and examination findings, laboratory indices, electrocardiogram (ECG) data, echocardiography data, angiography data, the status of the patient upon discharge (alive or dead), the date of subsequent admission, and discharge oral medication (Supplementary material). In our institution, heart failure patients are routinely prescribed guideline-directed medical therapy.

After exporting data from the PMRCardio database, data were merged into a single Microsoft Excel Sheet and imported into Python software, version 3.10.0. The international classification of diseases, tenth revision (ICD-10) code, was used to select patients diagnosed with heart failure due to any aetiology. The following ICD-10 diagnoses were used to select patients included in the analysis: “heart failure unspecified,” “congestive heart failure,” “left ventricular failure,” “dilated cardiomyopathy,” or “ischaemic cardiomyopathy.” Only heart failure patients 18 years of age and older with a left ventricular ejection fraction (LVEF) < 50% hospitalised between 2009 and 2018 were included in the dataset used to build predictive models. The rationale for only including patients with a LVEF < 50% is that in our institution, patients with a LVEF between 40 and 49% (*heart failure with a mid-range ejection fraction)* are managed similarly to those with LVEF < 40%.

All retrospective patient data used in the final analysis was further prospectively verified by comparing hard copies of medical records with electronic data. Patients without a documented LVEF were excluded from the analysis. Biochemical data captured at the time of admission was obtained from the National Health Laboratory Service (NHLS). Approval to conduct the study was received from the University of the Witwatersrand Human Research Ethics Committee (Clearance certificate number: M190515). Permission to conduct the study was also obtained from NHLS senior authorities. Informed consent was not obtained from the patients as this was a retrospective chart review. The study protocol conformed to the ethical guidelines of the 1975 Declaration of Helsinki as reflected in *a priori* approval by the institution’s human research committee.

### 2.2. Data cleaning and pre-processing

Packages and libraries required for data analysis were imported into Python software, version 3.10.0. The following software, packages and libraries were installed: Jupyter notebook version 6.0.3, scikit-learn version 1.02, statsmodels version 0.13.2, seaborn version 0.11.2, numpy version 1.21.6, scipystats version 1.5.4, matplotlib version 3.1.3, and pandas version 1.3.5. Features with more than 20% missing values, patient identifiers, and column data without variation were removed from the dataset. The remaining continuous features with missing data were imputed using the mean or median value. Categorical features were coded as 0 for no and 1 for yes, while blank or missing values were ascribed with zero. Data used to train machine learning algorithms was normalised, transformed and scaled between 0 and 1. The rationale for scaling the data is to standardise the weight of each feature. For example, sodium levels equal to 120 will have more weight than a potassium level of 5.0.

### 2.3. Exploratory data analysis and hyperparameter optimisation

Exploratory data analyses were carried out visually and quantitatively. Data were partitioned into training and test datasets, with 70% of the data used for training. Grid searching was implemented for all machine learning models, where parameters associated with the best performance metrics are discovered and selected during model hyper-tuning. Since the data is unbalanced with more patients in the survivors’ class, cost-sensitive learning was applied at a ratio of 0.14: 0.86, where a higher weight was allocated to the minority class (*dead = 1*).

All demographic and clinical parameters captured during arrival at the hospital were used as features. A filter method was then used to identify features that are positively or negatively correlated with all-cause mortality. Filtering was done using correlation coefficients. To ascertain feature-to-target correlation, a correlation coefficient was estimated for each feature. The threshold was set at 0.5. The lowest and highest values were furosemide (*r* = −0.277395, *p* < 0.001) and potassium (*r* = 0.180126, *p* < 0.001). Since all features were weakly correlated with the target value, none of the features were removed. To build predictive models, we only used features captured during the first hospitalisation. As such, the model with the best performance metrics will be used to predict the risk of in-hospital mortality using patient data obtained at the time of arrival at the cardiology wards at the CMJAH.

In this study, the prediction window is 4 years (interquartile range: 2–6 years). The prediction window starts from the time of hospitalisation (index date), where the risk of death is assessed, and ends at the time when a patient dies while hospitalised. The EHR system only stores a single entry of data. As such, there is no time window. For example, although clinical parameters are measured several times while the patient is hospitalised, only the first measurement (*baseline*) was captured in the EHR system. The parameters presented in the paper were collected from the baseline index admission for each of the 500 patients. The following six algorithms were used to build models predicting all-cause in-hospital mortality.

#### 2.3.1. Decision trees

Decision trees implement a sequential decision process based on whether the conditions set are true or false. The decision tree algorithm was trained, and the tree was plotted based on grid search results by placing the criterion as Gini, maximum depth at five, and the minimum sample split at eight.

#### 2.3.2. Random forest

A random forest classifier uses multiple decision trees, and each of the decision trees outputs a prediction (dead vs. alive). To estimate the final output, the random forest algorithm then counts the number of votes for each class predicted by each decision tree. Grid search identified the following parameters to be responsible for best model performance: the number of estimators = 1 757, minimum samples split = 5, minimum samples leaf = 2, maximum depth = 150, and criterion = entropy.

#### 2.3.3. Support vector machines

Support vector machines classify data by creating a line or hyperplane between the classes. The maximum distance between the classes (support vectors) and the hyperplane is then chosen. After applying grid search, the best parameters for creating a model were a linear kernel, gamma = 0.001, and *C* = 10. The “C” is a parameter that controls the effect of support vectors.

#### 2.3.4. Logistic regression

For the machine learning logistic regression model, the coefficient of each feature was obtained after training and testing models’ performance. A conventional statistical univariate and multivariate logistic regression model was also built to compare predictors of mortality identified by machine learning algorithms with those extracted from the statistical model. Clinical parameters with a *p*-value less than 0.05 were selected and included in the final multivariate logistic regression model to identify independent predictors of all-cause mortality. Confidence interval percentiles were set between 0.025 and 0.975.

#### 2.3.5. Extreme gradient boosting

Extreme gradient boosting is an ensemble of weak algorithms; usually, decision trees added to the model sequentially. Feature importance is calculated based on the number of times the feature appears in isolated trees.

#### 2.3.6. Multilayer perceptron

The MLP network consists of one input layer, hidden layers, and an output layer. Grid search was used to find the best model parameters. The search for optimal parameters was performed with the following limits: 0–0.4 dropout rate, batch size between 10 and 60, and epochs between 1 and 150, and the activation function was chosen as the rectified linear unit (ReLU). The learning rate was set at 0.01. The hyperparameters resulting in the best performing networks were as follows: a multi-layer network with 124 nodes using the ReLU activation function, batch size of 60 and 20 epochs. Shapley additive explanations (SHAP) values were used to identify features influencing the construction of the model.

Six supervised machine learning algorithms were trained to predict all-cause in-hospital mortality in heart failure patients with a LVEF less than 50% using 123 features. The performance metrics of each algorithm were summarised using the confusion matrix to calculate the accuracy, recall, precision, and F1 score, except for the MLP, where only the accuracy was reported since the rest of the performance metrics are not routinely available after training MLP.

Features identified by the algorithms were ranked, weighed and compared with predictors identified by logistic regression using the conventional statistical method. An area under the receiver operating characteristic (ROC) curve was used to evaluate the ability of each model to discriminate between the negative (*alive = 0*) and positive (*dead = 1*) classes.

### 2.4. Biostatistics

Categorical data are reported as absolute numbers and percentages, while the mean with standard deviation (±) and median with 25–75th percentile interquartile ranges were used to summarise numerical data with a normal and non-normal distribution, respectively. Normality was assessed visually with histograms and quantitatively with the Shapiro–Wilk and skewness tests. The Pearson chi-square and unpaired *t*-tests were used to compare categorical and normally distributed numerical data, respectively. In contrast, the Wilcoxon signed-rank test was used to compare numerical data with a non-normal distribution between patients that survived and died. A *p*-value less than 0.05 represented a statistically significant difference in the distribution of the features between survivors and non-survivors.

## 3. Results

### 3.1. Demographic and clinical findings

**FIGURE 1:**
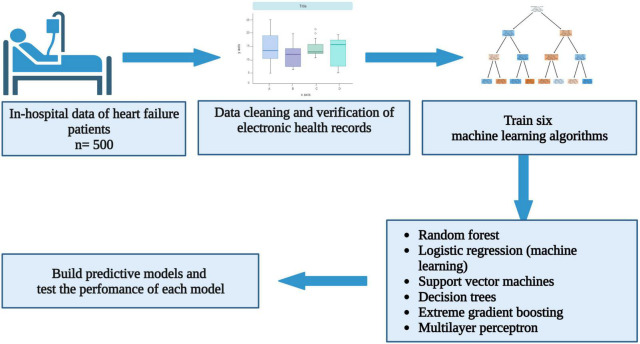
Flow chart showing study method and outcomes.

Between January 2009 and December 2018, there were 4,730 acute and chronic heart failure-related hospitalisations. After excluding patient records with missing LVEF and data that could not be verified using original hand-written patient records and biochemical data captured in the NHLS website, a total of 500 heart failure patients with a LVEF < 50% were included in the descriptive cohort analysis (Figure 1). The mean age was 55.2 ± 16.8 years, and 271 (54.2%) of these patients were males. The mean LVEF was 29 ± 9.2%. In this cohort, there were 300 (60.0%), 124 (24.8%), 54 (10.8%), and 22 (4.4%) Black, White, Indian, and Mixed ancestry patients, respectively. The rest of the baseline clinical characteristics of the study cohort are depicted in Table 1.

**TABLE 1:** Baseline demographic and clinical characteristics of all heart failure patients.

Clinical parameters	All patients (*n* = 500)	Died (*n* = 84)	Alive (*n* = 416)	*P*-value
Age (years)	55.2 ± 16.8	57.3 ± 18.6	54.8 ± 16.6	0.2162
Male	271 (54.2)	47 (55.9)	224 (53.8)	0.1249
Vital signs				
Pulse, bpm	102 ± 23.8	95 ± 25.5	103 ± 23.5	0.1963
Systolic BP, mmHg	127 ± 30.4	121 ± 31.7	128 ± 30.1	0.0588
Diastolic BP, mmHg	83 ± 20.4	83 ± 21.2	84 ± 20.3	0.6691
Laboratory tests				
Haemoglobin, g/dL	13.1 ± 2.5	13.3 ± 2.2	13.1 ± 2.5	0.4467
Sodium, mmol/l	139 ± 5.5	139 ± 5.5	139 ± 5.5	0.5220
Potassium, mmol/l	4.3 (3.9–4.7)	4.4 (3.9–4.7)	4.3 (3.9–4.7)	0.6610
Urea	8.6 (6.2–12.5)	8.05 (6–11.9)	8.7 (6.2–12.7)	0.2874
Creatinine	104 (80–137)	98 (81–124)	105 (80–142)	0.3774
eGFR, ml/min	65.7 (44.5–85.0)	68.0 (37.0–84.8)	65.4 (46.0– 85.0)	0.6116
Pro BNP, ng/l	8,541 (4,813–14,780)	7,592 (4,402–12,734)	8,709 (4,813–14,998)	0.3267
Troponin	23.5 (0.15–66.0)	4.06 (0.054–77.0)	24.5 (0.17–65.0)	0.3427
Total cholesterol	3.5 (2.8–4.1)	3.3 (2.8–4.1)	3.5 (2.8–4.2)	0.9705
LDL	2.1 (1.5–2.7)	2.1 (1.7–2.5)	2.1 (1.5–2.7)	0.9618
HDL	0.85 (0.6–1.1)	0.9 (0.6–1.2)	0.8 (0.6–1.1)	0.6493
Echocardiogram				
LVEF, %	29 ± 9.2	30 ± 9.2	29 ± 9.2	0.7356
LVIDd, cm	5.9 ± 1.0	5.9 ± 0.1	5.8 ± 0.9	0.8655
LVIDs, cm	5.1 (4.5–5.6)	5.0 (4.3–5.7)	5.1 (4.5–5.6)	0.5306
Length of stay (days)	7 (4–11)	7 (4–10)	7 (4–11)	0.5796

Data are represented as mean and standard deviation (SD) for continuous features with a normal distribution and median and interquartile ranges (p25–p75) when the distribution is skewed. Categorical data are represented as absolute numbers and percentages. BP, blood pressure; eGFR, estimated glomerular filtration rate; HDL, high-density lipoprotein; LDL, low-density lipoprotein; LVEF, left ventricular ejection fraction; LVIDd, left ventricular internal diameter at diastole; LVIDs, left ventricular internal diameter at systole.

There were 159 (31.8%) patients with hypertension and 268 (53.6%) patients with dilated cardiomyopathy. A total of 209 (41.8%) patients had hepatomegaly, 110 (22.0%) had ascites, 350 (55.0%) had mitral regurgitation, 340 (68.0%) had bipedal oedema, and 395 (79.0%) had an elevated jugular venous pressure on clinical examination. The mean resting pulse rate was 102 ± 23 beats per minute. Upon discharge from the hospital, there were 299 (59.8%) patients on oral beta-blocker therapy, and angiotensin-converting enzyme (ACE) inhibitors, furosemide and spironolactone were taken by 201 (40.2%), 300 (60.0%), 362 (72.4%) patients, respectively.

### 3.2. Outcomes

In the selected 500 patients hospitalised with heart failure, 84 (16.8%) patients died before discharge from the hospital. The median duration of hospitalisation was 7 days (interquartile range: 4–11), and it did not differ between patients discharged alive and those who died. Among the 416 patients discharged alive, 29 (7.0%) patients were readmitted within 30 days, 42 (10.1%) beyond 30 days, and 340 were not readmitted.

### 3.3. Performance metrics

**FIGURE 2:**
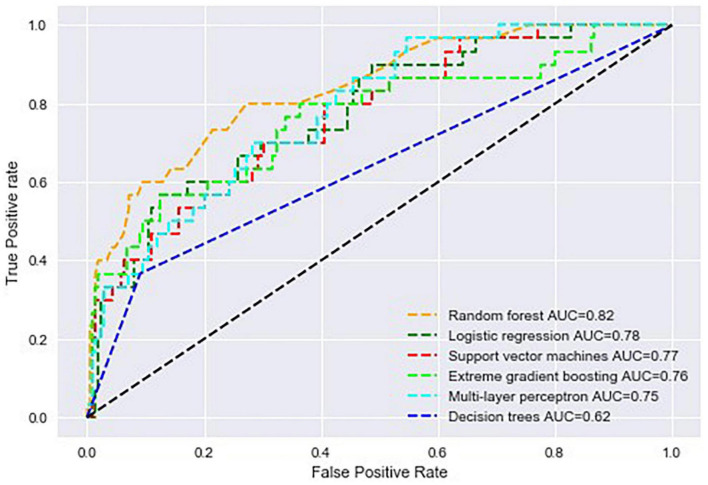
Area under the receiver operating characteristic curve of all machine learning algorithms used to build predictive models in the study.

Multilayer perceptron had an accuracy of 87% during training and testing. The AUROC curve was 0.82, 0.78, 0.77, 0.76, 0.75, and 0.62 for random forest, logistic regression (ML), SVM, XGBoost, MLP, and decision trees, respectively (Figure 2). Support vector machines achieved an accuracy of 86% during the test phase, followed by XGBoost, decision trees and logistic regression at 82, 78, and 76%, respectively (Table 2). The recall rate was above 50% in all algorithms, except for SVM, with a recall rate of 44 and 43%, respectively, during training and testing using data of patients that died while hospitalised. Logistic regression had the lowest precision of 39 and 29% during training and testing.

**TABLE 2:** Performance of supervised machine learning algorithms in all-cause mortality prediction on the PMRCardio dataset.

Algorithm	Accuracy	Precision	Recall	f1-score
**Random forest**				
Training: Survived	1.00	1.00	1.00	1.00
Mortality		1.00	1.00	1.00
Test: Survived	0.88	0.89	1.00	0.94
Mortality		0.75	0.10	0.18
Weighted training score		1.00	1.00	1.00
Weighted test score		0.87	0.88	0.84
**Logistic regression (ML)**				
Training: Survived	0.78	0.96	0.78	0.86
Mortality		0.39	0.83	0.53
Test: Survived	0.76	0.94	0.78	0.85
Mortality		0.29	0.63	0.40
Weighted training score		0.88	0.78	0.81
Weighted test score		0.86	0.76	0.79
**Support vector machines**				
Training: Survived	0.91	0.91	0.99	0.95
Mortality		0.92	0.44	0.60
Test: Survived	0.86	0.92	0.92	0.92
Mortality		0.45	0.43	0.44
Weighted training score		0.91	0.91	0.90
Weighted test score		0.86	0.86	0.86
**Extreme gradient boosting**				
Training: Survived	0.85	0.94	0.89	0.91
Mortality		0.50	0.67	0.57
Test: Survived	0.82	0.92	0.87	0.90
Mortality		0.36	0.50	0.42
Weighted training score		0.88	0.85	0.86
Weighted test score		0.85	0.82	0.84
**Decision trees**				
Training: Survived	0.86	0.99	0.84	0.91
Mortality		0.50	0.94	0.66
Test: Survived	0.78	0.95	0.79	0.86
Mortality		0.32	0.70	0.44
Weighted training score		0.92	0.86	0.87
Weighted test score		0.87	0.78	0.81

ML, machine learning. Performance metrics for the multilayer perceptron (MLP) model not included as the metrics reported in this table are not routinely generated with MLP model.

### 3.4. Feature ranking

#### 3.4.1. Multilayer perceptron

**FIGURE 3:**
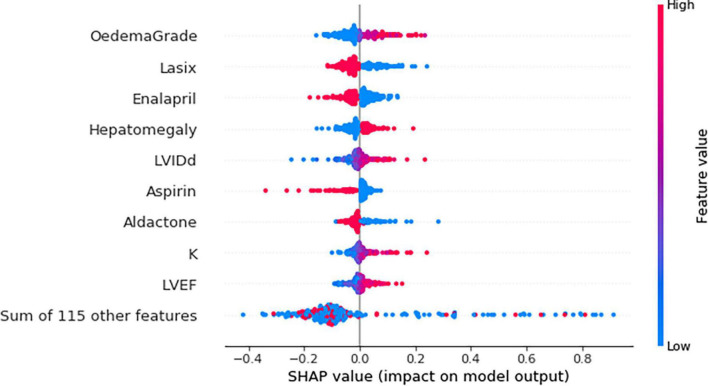
Beeswarm plot showing the impact of top nine feature on the multilayer perceptron (MLP) model output based on Shapley additive explanations (SHAP) values. K, potassium; LVEF, left ventricular ejection fraction; LVIDd, left ventricular internal diameter in diastole. Each dot represents a single patient. Red and blue refer to a high and low feature value, respectively. For example, both high and low potassium levels were associated with the risk of death. However, higher potassium levels (red) were strongly associated with the risk of mortality.

The top nine features identified by the MLP model were peripheral oedema grade, furosemide, enalapril, hepatomegaly, LVIDd, aspirin, spironolactone, serum potassium, and the LVEF (Figure 3).

#### 3.4.2. Logistic regression (ML)

**FIGURE 4:**
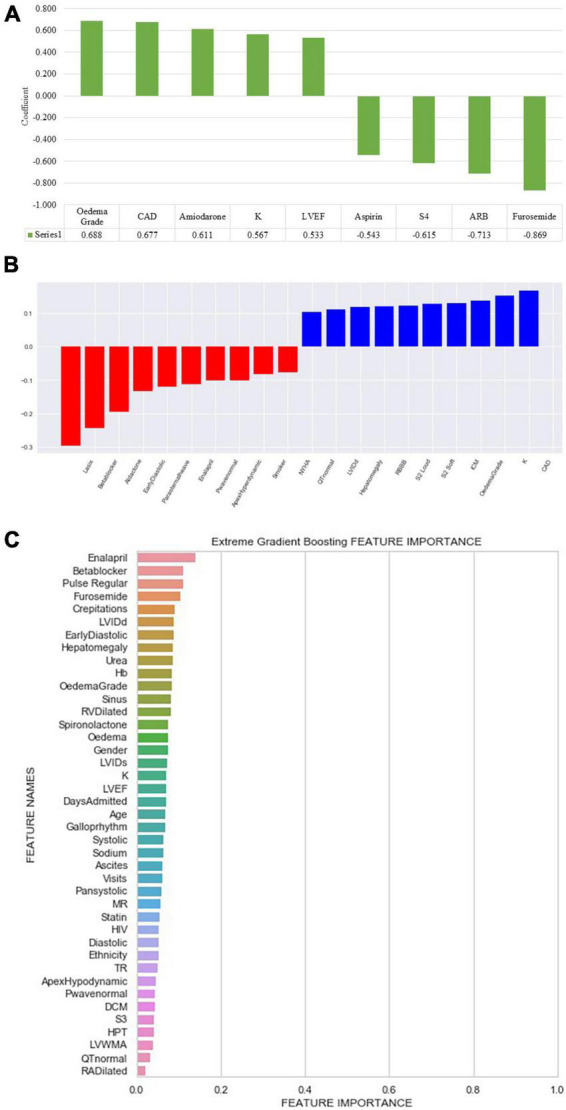
Feature importance and weight identified by machine learning algorithms. **(A)** Logistic regression (ML): ARB, angiotensin receptor blocker; CAD, coronary artery disease; K, potassium; LVEF, left ventricular ejection fraction; S4, fourth heart sound. **(B)** Support vector machine: CAD, coronary artery disease; ICM, ischaemic cardiomyopathy; K, potassium; LVIDd, left ventricular internal diameter in diastole; NYHA, New York Heart Association; RBBB, right bundle branch block; S2, second heart sound; Early diastolic, early diastolic murmur. **(C)** Extreme gradient boosted trees (XGB) classifier ranked features based on how many times a feature is used to split the data across all trees. Crepitations, lung crepitations on chest auscultation; Days admitted, duration of hospitalisation; DCM, dilated cardiomyopathy; Diastolic, diastolic blood pressure; Early diastolic, early diastolic murmur; Hb, haemoglobin; HIV, human immunodeficiency virus; HPT, hypertension; LVEF, left ventricular ejection fraction; LVIDd, left ventricular internal diameter in diastole; LVIDs, left ventricular internal diameter in systole; LVWMA, left ventricular wall motion abnormality; MR, mitral regurgitation; Pan systolic, pan systolic murmur; RA, right atrium; RV, right ventricle; S3, third heart sound; Sinus, sinus rhythm; Systolic, systolic blood pressure; TR, tricuspid regurgitation; visits, number of hospitalisations.

Peripheral oedema grade had a coefficient of 0.688. Furosemide, angiotensin receptor blockers, a fourth heart sound on auscultation and aspirin, negatively correlated with all-cause mortality (Figure 4).

#### 3.4.3. Support vector machines

The following top five features had a positive coefficient with the target feature: furosemide, beta-blockers, spironolactone, early diastolic murmur, and a parasternal heave, whereas coronary artery disease, potassium, oedema grade, ischaemic cardiomyopathy, and right bundle branch block on electrocardiogram had negative coefficients. The rest of the features are depicted in Figure 4.

#### 3.4.4. Extreme gradient boosting

Enalapril was the most common feature used to split decision trees and was used 20 times, followed by oral beta-blocker therapy, elevated jugular venous pressure, furosemide, and CAD (Figure 4).

#### 3.4.5. Decision trees

Decision trees identified beta blockers as the root node (Gini = 0.5). In addition, the following features were placed as leaf nodes: systolic and diastolic blood pressure, urea, duration, the number of hospitalisations, and CAD.

#### 3.4.6. Random forest

**FIGURE 5:**
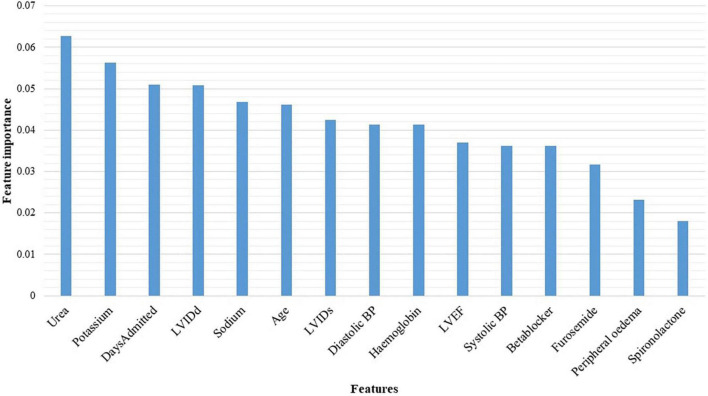
Features identified by the random forest algorithm. BP, blood pressure; LVEF, left ventricular ejection fraction; LVIDd, left ventricular internal diameter in diastole; LVIDs, left ventricular internal diameter in systole.

Urea, potassium, duration of admission, LVIDd, serum sodium levels, age, LVIDs, diastolic blood pressure, haemoglobin, and the LVEF, were identified as the top 10 features discriminating the two classes (Alive vs. Dead) (Figure 5).

#### 3.4.7. The conventional statistical logistic regression model

In the multivariate logistic regression model, the following variables were independent predictors of all-cause mortality: diastolic blood pressure, coronary artery disease, enalapril, spironolactone, furosemide, sodium, and potassium (Table 3).

**TABLE 3:** Conventional statistical multivariate logistic regression model predicting all-cause mortality.

	Univariate regression			Multivariate regression		
	**Coefficient**	***P*-value**	**CI**	**Coefficient**	***P*-value**	**CI**
Female	0.4330	0.043	0.013–0.853			
Black race	0.5169	0.028	0.055–0.978			
Gallop rhythm	0.5399	0.045	0.012–1.068			
ED murmur	-0.5496	0.012	−0.977 to 0.122			
Diastolic BP	-0.0356	0.000	−0.049 to −0.022	-0.0239	0.002	−0.039 to −0.009
CAD	0.7432	0.022	0.105–1.381	0.9490	0.033	0.078–1.820
Spironolactone	-1.0043	0.000	−1.412 to 0.596	-0.6674	0.012	−1.190 to −0.145
Enalapril	-1.3472	0.000	−1.845 to 0.849	-0.6440	0.044	−1.270 to −0.018
Aspirin	-0.6101	0.037	−1.183 to −0.037			
Furosemide	-1.5959	0.000	−2.022 to −1.170	-1.5685	0.000	−2.142 to −0.995
Statins	-0.6847	0.012	−1.216 to −0.153			
Oedema grade	0.2503	0.002	0.089 to 0.411			
Sodium	-0.0782	0.000	−0.115 to −0.041	-0.0288	0.001	−0.045 to −0.012
Potassium	0.6609	0.000	0.398–0.924	0.5366	0.001	0.230–0.843
Urea	0.0095	0.032	0.001–0.018			
RBBB	0.9321	0.007	0.259–1.606			
LVIDd	0.3835	0.000	0.175–0.592			
LVIDs	0.3337	0.002	0.122–0.545			
Length of stay (days)	0.0257	0.012	0.006–0.046			

CAD, coronary artery disease; BP, blood pressure; CI, confidence interval (0.025–0.975); ED, end diastolic; RBBB, right bundle branch block; LVIDd, left ventricular internal diameter in diastole; LVIDs, left ventricular internal diameter in systole.

## 4. Discussion

This study used data from 500 patients with a LVEF less than 50% on the index or baseline hospitalisation to train six supervised machine learning algorithms to predict all-cause mortality. Although the random forest algorithm achieved a higher AUROC curve, the striking difference in training and testing performance metrics suggests that the model is overfitting data. Support vector machines had an AUC of 0.77, and an accuracy of 91 and 86% during training and testing, making it an ideal model for validation. The most plausible explanation for the SVM showing a desirable performance lies in the ability of the algorithm to process and model complex and non-linear data by creating a decision boundary that separates classes.

The all-cause mortality and 30-day rehospitalisation rate in 500 patients admitted with heart failure were 16.8 and 7.0%, respectively, while the median duration of hospitalisation was 7 (interquartile range: 4–11) days. In another tertiary-level hospital in South Africa, the in-hospital mortality rate among patients with acute heart failure was 8.4% and the mean duration of hospitalisation was 9 ± 12 days (1). Similarly, in a multicentre study conducted across Africa involving 169 heart failure patients recruited in South Africa, the 1-year all-cause mortality was 11.8% (2). In a meta-analysis involving 67,255 heart failure patients hospitalised in Australia, the pooled 30-day all-cause mortality was 8% (3). Similarly, in another systematic review of research studies conducted in low-and-middle-income countries (LMIC), the in-hospital all-cause mortality was 8%, with a confidence interval of 6–10% (4). The most plausible explanation for our cohort’s higher in-hospital all-cause mortality is the delay in presentation to the hospital. As a tertiary academic centre, most patients referred to our centre are initially managed in primary and secondary level institutions and eventually referred to our centre in advanced stages of heart failure.

Despite the variation in the aetiology of heart failure between patients residing in LMIC and HIC, and limited access to life-prolonging intracardiac device therapy, particularly in patients attending state-owned treatment facilities, we found similar predictors of all-cause mortality using machine learning algorithms and a conventional statistical logistic regression model. After training the SVM, furosemide, beta-blockers, spironolactone, early diastolic murmur and a parasternal heave, yielded positive coefficients, whereas coronary artery disease, potassium, oedema grade, ischaemic cardiomyopathy, and right bundle branch block on electrocardiogram had negative coefficients. The support vector machines and the machine learning logistic regression algorithm identified similar predictors as the MLP model. Some of the predictors of all-cause mortality in heart failure reported in the literature include age, sex, diastolic blood pressure, LVEF, serum sodium and creatinine, estimated glomerular filtration rate, haemoglobin, haematocrit, and blood urea nitrogen (5–7). Predictors of mortality specifically in patients with a LVEF above 50% include blood urea nitrogen and the body mass index (6).

Healthcare databases are notoriously famous for housing imbalanced datasets. Class imbalance refers to the disproportionality between the data classes used to train the predictive model (8), a common problem that is not unique to medical data. For example, when the training data with the negative outcome (*dead*) has significantly fewer observations than the majority class, the classification algorithm favours the majority class, by focusing the learning process on the majority class. Our dataset had fewer entries attributed to the negative outcome and was imbalanced. This accounts for the suboptimal performance shown by the algorithms when learning and making predictions specifically for the minority class. However, instead of under-sampling the majority class or generating new synthetic values to increase the number of samples in the minority class, we used class weights to excessively penalise false negative and false positive results from the minority class (*dead*).

Six supervised machine learning algorithms were successfully trained to predict mortality despite an imbalanced and relatively small dataset. The success implies that data quality is also essential to consider before building predictive models. The project’s next phase is to increase the sample size by collecting data from multiple cardiac centres around sub-Saharan Africa. Adding new data that was not previously used to train or test the algorithms tends to improve the models’ performance metrics. Once the performance of the classifier has been validated, a risk calculator will be developed and implemented for use in Africa.

The performance of machine learning algorithms is commonly attributed to the size of the dataset. Indeed, the higher the number of observations the better the performance metrics. Some authors have attempted to derive an equation for calculating a suitable sample size for machine learning algorithms by calculating the error rate of prediction (9, 10). In our opinion, the performance of machine learning algorithms is also influenced by the number of features (clinical parameters) used, the ratio of observations within the classes, and the credibility of the data. In our study, 500 patients with 123 features were used to train algorithms. Consider a model created with over a million patients, but with only five features. Its performance might be suboptimal. An ideal sample size calculation should incorporate the number of observations and features and a measure of data credibility. In general, the error rate should decrease as the size of the training dataset increases. The AUROC curve for the machine learning models can be improved by increasing the size of the training dataset. Other techniques for improving the AUC such as feature normalization and scaling, setting class weights, and grid search were applied in this study.

Machine learning algorithms cannot process data with empty fields. As such innovative ways of handling missing data have been introduced. These include deleting incomplete records, filling incomplete fields with the mean, median or mode, and predicting missing values using machine learning algorithms and applying the last observation carried forward method (11). In this study, we removed clinical parameters with more than 20% missing values and imputed missing values using the mean or median value of each feature. The rationale for using the mean is that overall the mean value (Gaussian distribution) of each feature does not change. However, this method does not factor for the covariance between features.

This was a single-centre study conducted in a tertiary centre since the CMJAH division of cardiology is the only department equipped with an electronic health record system. As such, the findings from this study may not be generalisable to all heart failure patients treated in SSA. In addition, most pertinent clinical parameters such as comorbidities, causes of heart failure, angiographic, and ECG findings were available in the database but underreported, as some of the data were captured in the free text format and could not be extracted for analysis and incorporation into the predictive models. Also, we excluded a significant amount of patient data due to the unavailability of handwritten patient records or objective clinical evidence supporting the heart failure diagnosis. This was a major limitation, since handwritten patient records are routinely removed from the CMJAH after 5 years from the time of initial admission, due to the unavailability of storage facilities. Furthermore, pertinent clinical data, such as the presence of cardiogenic shock, and the administration of ionotropes and vasopressors, were documented in the EHR system in free-text format and could not be extracted. Nevertheless, the study provides proof of concept for developing a heart failure model derived from LMIC predicting all-cause mortality.

## 5. Conclusion

Despite a small sample size, machine learning algorithms successfully predicted all-cause mortality with modest accuracy. The SVM model will be externally validated using data from multiple cardiology centres in South Africa before developing a uniquely African risk prediction tool.

## Data availability statement

The raw data supporting the conclusions of this article will be made available by the authors, without undue reservation.

## Ethics statement

The studies involving human participants were reviewed and approved by University of the Witwatersrand Human Research Ethics Committee (Clearance certificate number: M190515). Written informed consent for participation was not required for this study in accordance with the national legislation and the institutional requirements.

## Author contributions

DM: conceptualization, data curation, formal analysis, funding acquisition, investigation, methodology, project administration, resources, software, validation, visualization, roles/writing—original draft, and writing—review and editing. TC: conceptualization, data curation, formal analysis, funding acquisition, investigation, methodology, project administration, resources, software, supervision, validation, visualization, roles/writing—original draft, and writing—review and editing. EK: methodology, validation, roles/writing—original draft, and writing—review and editing. HN: conceptualization, data curation, formal analysis, funding acquisition, investigation, methodology, project administration, resources, supervision, validation, visualization, roles/writing—original draft, and writing—review and editing. All authors contributed to the article and approved the submitted version.
