# Supplementary material for: Predicting in-hospital all-cause mortality in heart failure using machine learning
Source: Front Cardiovasc Med. 2023 Jan 11;9:1032524. doi: 10.3389/fcvm.2022.1032524 (PMC9875063; doi:10.3389/fcvm.2022.1032524)
Supplement: Supplementary file 1 [file Table_1.DOCX]

**Supplementary file 1**

Clinical parameters extracted from the Electronic Health Record system

|  | **Categorical variables** | **Continuous variables** |
| --- | --- | --- |
| **Demographic data** | Gender, ethnicity, international classification of diseases, tenth revision (ICD-10) code, | Age |
| **Co-morbidities** | Hypertension, diabetes mellitus, smoking, dyslipidaemia, family history of coronary artery disease, human immunodeficiency virus (HIV) and chronic kidney disease |  |
| **Clinical examination findings** | Lung crepitations, ascites, elevated jugular venous pressure, pedal oedema, displaced apex beat, | Systolic and diastolic blood pressure |
| **Electrocardiography** | Rhythm, P wave, Q wave, bundle branch block, ST segment | Heart rate, PR interval, QT interval |
| **Echocardiography** |  | Left ventricular internal diameter at diastole (LVIDd), Left ventricular internal diameter at systole (LVIDs), Left ventricular ejection fraction (LVEF) and Left atrial size |
| **Diseased vessel on coronary angiography** | Left main artery, left anterior descending artery, right coronary artery, circumflex artery and diagonal artery |  |
| **Laboratory results** |  | Troponin I, haemoglobin, sodium, potassium, urea, creatinine, International Normalized Ratio (INR), total cholesterol, low-density lipoprotein (LDL), high-density lipoprotein (HDL), glucose, glycated haemoglobin (HBA1c) and pro-brain natriuretic peptide (proBNP) |
| **Oral medication** | Aspirin, beta-blockers, furosemide, angiotensin converting enzyme (ACE) inhibitors, calcium antagonists and statins |  |
| **Outcome** | Mortality  Hospitalisation | Length of hospital stay |
